# Impact of influenza vaccination in the Netherlands, 2007–2016: Vaccinees consult their general practitioner for clinically diagnosed influenza, acute respiratory infections, and pneumonia more often than non-vaccinees

**DOI:** 10.1371/journal.pone.0249883

**Published:** 2021-05-28

**Authors:** Saverio Caini, John Paget, Peter Spreeuwenberg, Joke C. Korevaar, Adam Meijer, Mariëtte Hooiveld

**Affiliations:** 1 Nivel (Netherlands Institute for Health Services Research), Utrecht, The Netherlands; 2 National Institute for Public Health and the Environment (RIVM), Bilthoven, The Netherlands; Health Directorate, LUXEMBOURG

## Abstract

**Introduction:**

We aimed to develop an innovative population-based method to estimate the health effect of influenza vaccination based on electronic medical records collected within a general practitioner (GP)-based influenza surveillance system in the Netherlands.

**Methods:**

In each season between 2006/07 and 2015/16, we fitted multilevel Poisson regression models to compare GP consultation rates for clinically diagnosed influenza, acute respiratory infections (ARI), pneumonia, and lower back pain (as a control) between vaccinated vs. unvaccinated individuals. Season-specific relative risks and 95% confidence intervals (CI) were pooled into summary risk ratio (SRR) through random-effects meta-analysis models. Analyses were stratified by patient age (<45, 45–59, 60–74, ≥75 years) and medical indication for the vaccine (any vs. none, subjects aged ≤60 years only).

**Results:**

Overall, 12.6% and 21.4% of study subjects were vaccinated because of their age only or because of an underlying medical condition. Vaccine uptake declined over time, especially among subjects aged ≤74 years with medical indications for vaccination. Vaccinated individuals had significantly higher GP consultation rates for clinically diagnosed influenza (SRR 1.24, 95% CI 1.12–1.38, p-value <0.001), ARI (SRR 1.33, 95% CI 1.27–1.39, p-value <0.001), pneumonia (SRR 1.27, 95% CI 1.19–1.36, p-value <0.001), and lower back pain (SRR 1.21, 95% CI 1.14–1.28, p-value <0.001) compared to unvaccinated individuals.

**Discussion:**

Contrary to expectations, influenza vaccinees have GP consultation rates for clinically diagnosed influenza, ARI and pneumonia that are 24–33% higher compared to unvaccinated individuals. The lower back pain finding suggests that the increase in consultation rates is partially caused by confounding. Importantly, considering the data are not laboratory-confirmed, our results cannot be linked directly to influenza, but only to respiratory illnesses in general.

## Introduction

Influenza is associated with a considerable global burden of disease, in terms of morbidity, mortality, and economic costs [1], and efforts to mitigate the impact of the disease on individuals, societies and healthcare systems are an important public health priority. Along with surveillance and preparedness, annual vaccination is the cornerstone of influenza prevention. Influenza vaccination is primarily recommended for those at greater risk of developing life-threatening complications: these include elderly people (i.e. aged ≥65 years) and people of any age suffering from certain chronic illnesses or medical conditions (e.g. heart disease, diabetes, asthma, severe obesity, and others) or living in long-term care facilities [2, 3].

The health impact of influenza vaccination campaigns depends on several factors, the most important of which are the vaccine uptake by the population subgroups targeted by the immunization campaigns, and the vaccine effectiveness (VE). The latter can be defined as the reduction of laboratory-confirmed influenza relative risk among vaccinated vs. non-vaccinated individuals as estimated from real-world observational studies [4]. In recent years, the test-negative design (TND) has established itself as the study design most widely adopted to monitor influenza VE annually [5, 6], although other designs (e.g. classical case-control studies and prospective cohorts) were also used. These studies mostly showed that influenza vaccines are effective in reducing the risk of being infected, although VE may fluctuate considerably across seasons [7–9], and appears to vary also depending on host-related characteristics (e.g. age, health status, and past history of vaccination [10–15]), virus type, subtypes and lineage [16], and other factors.

In the present paper, we aimed to develop an alternative method of estimating the health effect of influenza vaccination that is based on the use of electronic medical records (EMR) routinely collected within a general practitioner (GP)-based influenza surveillance system. Our objective was to develop a population-based method that could complement the findings from TND studies. Considering influenza vaccines are effective in preventing influenza infections (albeit generally moderately), our research hypothesis was that vaccine recipients would less often receive a clinical (i.e. not laboratory confirmed) diagnosis of respiratory illness (influenza, acute respiratory infection (ARI), or pneumonia) from their GP compared to non-vaccinated persons.

## Materials and methods

### Study design and data availability

This is an observational study based on data collected prospectively and in real-time in the Netherlands (northern Europe, population ≈ 17 million) during ten consecutive influenza seasons, from 2006/07 to 2015/16. In the Netherlands, seasonal influenza vaccination is provided free of charge to persons with a medical indication (e.g. cardiovascular diseases, pulmonary diseases, diabetes mellitus) regardless of age, and to all persons above a certain age (65 years up to 2007, 60 years thereafter) [17].

Data were obtained from Nivel Primary Care Database (Nivel PCD) (https://www.nivel.nl/en/nivel-primary-care-database). The number of participating general practices increased from about 80 in 2006, covering a representative sample of about 2% of the total population, to about 500 in 2016, covering about 10% of the population. The GP practices included in the Nivel PCD are geographically distributed throughout the country and representative of the age distribution of the Dutch population [18]. For this study, which included a subset of GPs and patients, there was a slight difference in the regional distribution of patients (a small over-representation of patients from the North, and an under-representation of patients from the West) but, importantly, the age distribution of patients was still very similar to that of the Dutch population. Pseudonymised data included morbidity and drug prescriptions of all patients enlisted in these practices, together with patients’ demographics. Diagnoses were recorded and classified by general practitioners according to the International Classification of Primary Care 1 (ICPC-1) [19], while prescriptions were coded according to the Anatomical Therapeutic Chemical (ATC) classification [20]. Only data from practices meeting strict quality criteria were included in the analysis: complete data of the year of vaccination and the subsequent calendar year, defined as at least 46 weeks per year, and a complete registration of influenza vaccination, defined as having records of vaccinated persons for at least 80% of the number of claimed vaccines. The number of claimed vaccines is provided by the National Influenza Prevention Programme Foundation that coordinates and organizes the vaccination campaign. The 80% is taking into account an annual average 10% mobility of the practice population, 5% vaccine spillage and 5% other reasons. Previous research showed that using at least 80% matching with the number of claimed vaccines provided good estimates of vaccination coverage [21].

We collected the weekly population aggregated number of new episodes of clinically diagnosed (i.e. not laboratory confirmed) influenza and ARI, and pneumonia (also clinically diagnosed, i.e. not confirmed by X-ray, CRP test or alike) in vaccinated and non-vaccinated patients, along with population denominator [22]. The vaccination status was defined as “non-vaccinated” if the person received the vaccine less than 14 days before the date of clinical diagnosis. The Dutch College of General Practitioners has defined a set of diagnoses and prescriptions codes to select persons with a medical indication for vaccination. We looked for these codes in records up to 18 months before the vaccination campaign (mid-October to mid-November) [21]. In addition to influenza, ARI, and pneumonia, we aimed to replicate the analyses using as the outcome a “control diagnosis”, i.e. a health outcome whose incidence rates are not expected to be affected by the vaccination status. The rationale was that for such an outcome, any differences in GP consultation rates between vaccinated and unvaccinated subjects would be the consequence of factors other than the vaccine. Lower back pain was chosen over other potential control diagnoses because its incidence is non-negligible in all the age groups considered in our study. In order to specifically focus on recent episodes of lower back pain, we only considered patients who did not consult the GP for the same reason in the preceding 52 weeks. The choice of lower back pain as control diagnosis is a standard procedure for studies with this design [23, 24]; alternative diagnoses that were considered were discarded for different reasons, e.g. because of their low frequency (e.g. accidents), or because they would not lead to GP consultations in a high proportion of cases (e.g. gastroenteritis).

The use of electronic health records for research purposes is allowed under certain conditions. When these conditions are fulfilled, neither obtaining informed consent from patients nor approval by a medical ethics committee is obligatory for this type of observational studies containing no directly identifiable data in the Netherlands (art. 24 GDPR Implementation Act jo art. 9.2 sub j GDPR).

### Statistical analysis

In each season during the study period, we aimed to compare the risk of receiving a diagnosis of influenza, ARI, pneumonia, and lower back pain following consultation with a GP, between influenza vaccinees vs. non-vaccinees. This was carried out by calculating risk ratio (RR) and corresponding 95% confidence intervals (CI) using multilevel Poisson regression models with week and GP practice as first and second level, respectively. Models were estimated using the MLwiN 2.30 software by means of a restricted iterative generalized least squares algorithm with second order predictive quasi-likelihood and unconstrained Poisson error variance (to allow for over-dispersed error variance). In each model, the numerator and the denominator were the total number of subjects with an incident diagnosis, and the overall number of subjects, in that week per-GP practice.

Previous research has shown that the patient’s age and health status are important determinants of influenza VE [10–13, 25]. Therefore, RRs were calculated separately in each of six population subgroups defined by age (<45, 45–59, 60–74, ≥75 years) and, for those older than 60 years, the presence of medical indications for influenza vaccination (any vs. none). Patients younger than 60 years with no medical indication for the vaccination were not included, as these are not eligible for free vaccination by their GP. They can be offered the vaccination, e.g.by their employers, but then their vaccination status might not be registered in the Nivel PCD and cannot be reliably determined. The inclusion of multiple outcomes in the analysis was motivated by the intent to carry out an internal cross-check, comparing the RR obtained for influenza with that of illnesses whose risk is expected to be affected to a lesser extent than influenza (ARI and pneumonia) or not at all (lower back pain) by the vaccine.

Season-specific RRs were pooled into summary risk ratio (SRR) through random-effects models with maximum likelihood estimates [26]. The forest plots were generated using the forestplot package in R software (version 4.0.0). The variability of RRs among seasons was quantified using the I^2^ statistics, which can be interpreted as the percentage of total variation that is attributable to actual heterogeneity rather than chance [27]. The larger the I^2^, the greater the heterogeneity, and values of I^2^ above 50% are usually considered as not compatible with chance alone. When this occurred, attempts were made to identify epidemiological characteristics that could account for part of the observed heterogeneity. In detail, meta-regression and subgroup analyses were conducted (for continuous and categorical variables, respectively) to test whether the season-specific RRs differed depending on what was (or were) the “dominant” virus (sub)type(s), i.e. that (of those) that accounted for at least 80% of all influenza cases in the season, and on what percentage of total cases in a season were caused by viruses that were mismatched with the viral strains contained in the vaccine. For this purpose, we considered both lineage-level vaccine mismatches (influenza B) and vaccine mismatches by antigenic drift (influenza A and B) (for simplicity, we ignored the variability in titer difference in hemagglutination-inhibition assays between homologous and tests viruses, and, for B viruses, cross reactivity between Victoria and Yamagata lineages) [28]. A meta-regression model was also fitted to test for a linear association between the calendar year and the log(RR) (i.e. we tested whether the RR tended to increase or decrease over time during the study period).

## Results

### Study population and influenza epidemiology in the Netherlands in the study period

The study database encompassed information about individuals who were seen at a general practice in The Netherlands between the seasons of 2006/07 and 2015/16. The number of GPs participating in the surveillance scheme increased over time, from fewer than 20 in the first two seasons, to over 200 from 2013/14 onwards (Table 1). Likewise, the study population included in each season rose from just over 50,000 in the first two seasons, to a maximum of over one million in 2014/15, to decline to around 500,000 in 2015/16. Influenza A viruses caused the majority of laboratory-confirmed influenza cases in all seasons during the study period except in 2007–08, when 51% of cases were caused by the B/Yamagata virus lineage (Table 1). Some level of antigenic and/or lineage-level mismatch between circulating influenza viral strains and those contained in the vaccine was observed in the majority seasons during the study period (Table 1) [9, 29, 30].

**Table 1:** Number of general practitioners, population studied, and background virological information (circulating influenza viruses and vaccine mismatch) in each season. Netherlands, 2006/07 to 2015/16.

	2006/07	2007/08	2008/09	2009/10	2010/11	2011/12	2012/13	2013/14	2014/15	2015/16
**I. Nivel Primary Care Database**										
General practitioners in the study	16	18	24	32	51	103	144	222	268	209
No. of population included	52,989	55,191	85,439	118,251	193,459	364,756	510,505	812,208	1,026,795	755,210
**II. Virological data (sentinel and non-sentinel data)**										
**1. Proportion of virus (sub)types (%)**	** **	** **	** **	** **	** **	** **	** **	** **	** **	** **
**Influenza A**	**99%**	**49%**	**92%**	**100%**	**60%**	**90%**	**69%**	**94%**	**82%**	**67%**
A(H1N1) ^(a)^	12%	86%	2%	100%	97%	1%	58%	40%	9%	98%
A(H3N2)	88%	14%	98%	0%	3%	99%	42%	60%	91%	2%
**Influenza B**	**1%**	**51%**	**8%**	**0%**	**40%**	**10%**	**31%**	**6%**	**18%**	**33%**
B Victoria	NA	0%	100%	0%	95%	12%	6%	24%	1%	94%
B Yamagata	NA	100%	0%	0%	5%	88%	94%	76%	99%	6%
**2. Dominant virus (see criteria below)**^**(b)**^	A(H3N2)	A(H1N1) & B Yam	A(H3N2)	A(H1N1)	A(H1N1) & B Vic	A(H3N2)	A(H1N1) & A(H3N2) & B Yam	A(H3N2) & A(H1N1)	A(H3N2) & B Yam	A(H1N1) % B Vic
**3. Mismatch assessment**	** **	** **	** **	** **	** **	** **	** **	** **	** **	** **
B lineage in the vaccine	Victoria	Victoria	Yamagata	Victoria	Victoria	Victoria	Yamagata	Yamagata	Yamagata	Yamagata
Vaccine mismatch	No	Yes B Yam	No	Yes A(H1N1)	No	Yes A(H3N2)	Yes A(H3N2)	Yes A(H3N2)	Yes A(H3N2)	Yes B Vic
Vaccine mismatch (%) ^(c)^	0%	51%	0%	100%	0%	89%	29%	56%	75%	31%

^(a)^ A(H1N1) has been replaced by A(H1N1)pdm09 the season from 2009/10 onwards.

^(b)^ Dominant virus criteria, applied hierarchically (80% strict criteria):

- One circulating virus accounting for ≥80% of cases.

- Two viruses circulating and accounting for ≥80% of cases.

- Three viruses circulating and accounting for ≥80% of cases.

^(c)^ Calculated as the proportion of total cases that were accounted for by the mismatched virus subtype(s). See text (Methods) for details.

NA: not available.

About one third (34.0%) of the total GP population was made up of subjects who had an indication to receive the influenza vaccine because of their age only (12.6%) or an underlying medical condition (21.4%). The vaccine coverage among study subjects with a medical indication to vaccinate correlated with age (from 44.7% among those aged <45 years to 81.2% among those aged 75 years or older), and was higher than among study subjects of the same age groups with no medical indication for the vaccine (Table 2). By and large, the proportion of study subjects who were vaccinated declined over time, considerably among patients with a medical indication for the vaccine aged less than 74 years, and less so in the other subgroups (Table 2).

**Table 2:** Study subjects (denominators), and proportion of those who were vaccinated, in each season and overall, according to the subjects’ age and presence of a medical indication to vaccinate. Netherlands, 2006/07 to 2015/16.

Age group	Season										Total
	2006/07	2007/08	2008/09	2009/10	2010/11	2011/12	2012/13	2013/14	2014/15	2015/16	
**Subjects with medical indications to vaccination**											
**<45 years**											
N patients	3,219	3,093	5,267	8,018	11,427	20,345	22,248	32,259	43,671	31,164	180,711
% vaccinated	61.6%	57.9%	44.0%	61.2%	45.5%	44.5%	47.3%	48.7%	40.8%	36.9%	44.7%
**45–59 years**											
N patients	2,625	2,573	4,403	6,620	10,666	19,886	25,661	40,456	55,982	41,793	210,665
% vaccinated	77.4%	74.6%	62.2%	72.2%	63.3%	59.8%	58.7%	59.5%	52.1%	47.7%	56.2%
**60–74 years**											
N patients	2,972	3,158	5,251	8,403	14,603	25,320	33,145	49,416	72,762	58,688	273,718
% vaccinated	85.1%	84.5%	77.1%	82.6%	75.2%	69.8%	70.4%	71.3%	66.5%	63.7%	69.1%
**75+ years**											
N patients	1,774	1,931	3,194	5,100	8,716	15,606	22,764	34,259	49,826	38,253	181,423
% vaccinated	87.4%	86.9%	87.4%	90.6%	86.5%	81.1%	81.3%	81.9%	79.7%	78.7%	81.2%
**Subjects without medical indications to vaccination**^**(a)**^											
**60–74 years**											
N patients	4,166	5,012	7,581	9,980	14,623	32,289	50,025	84,271	103,952	78,706	390,605
% vaccinated	40.8%	38.9%	63.0%	68.9%	60.2%	54.2%	50.1%	47.4%	42.3%	38.7%	46.3%
**75+ years**											
N patients	1,550	1,720	2,369	3,052	4,680	9,563	15,140	23,747	29,012	20,929	111,762
% vaccinated	78.8%	77.7%	81.3%	79.1%	77.0%	72.7%	72.1%	71.8%	68.3%	65.2%	70.6%
**Total population in analysis**											
N patients	16,306	17,487	28,065	41,173	64,715	123,009	168,983	264,408	355,205	269,533	1,348,884
% vaccinated	67.5%	64.9%	66.3%	74.2%	66.3%	61.6%	61.2%	60.5%	56.0%	53.1%	59.0%

^(a)^ Persons aged <60 years without a medical indication for influenza vaccination were not included as these are often not vaccinated by their GP and, therefore, their vaccination status is not registered in the Nivel-PCD (see text for details)

### Impact of influenza vaccination on clinically diagnosed health outcomes

**Fig 1:**
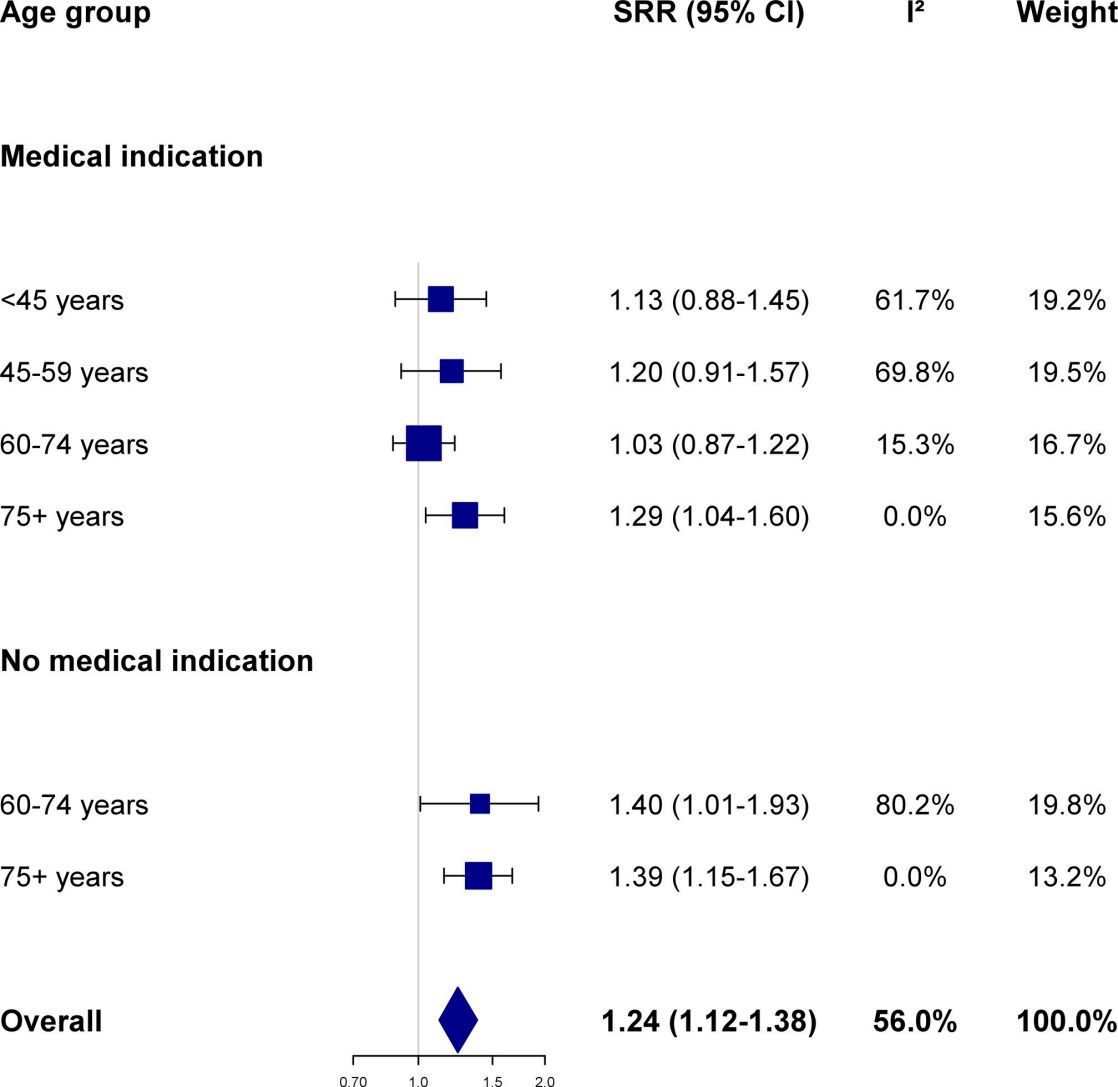
Association between influenza vaccination and the risk of influenza according to age group and presence of medical indication. Summary risk ratio (SRR) and 95% confidence intervals (CI) were calculated by pooling season-specific risk ratio using random effects meta-analysis models; between-seasons heterogeneity was quantified using the I^2^ statistics (see text for details; season-specific risk ratio were provided in S1 Table). The Netherlands, seasons 2006/07 to 2015/16.

**Fig 2:**
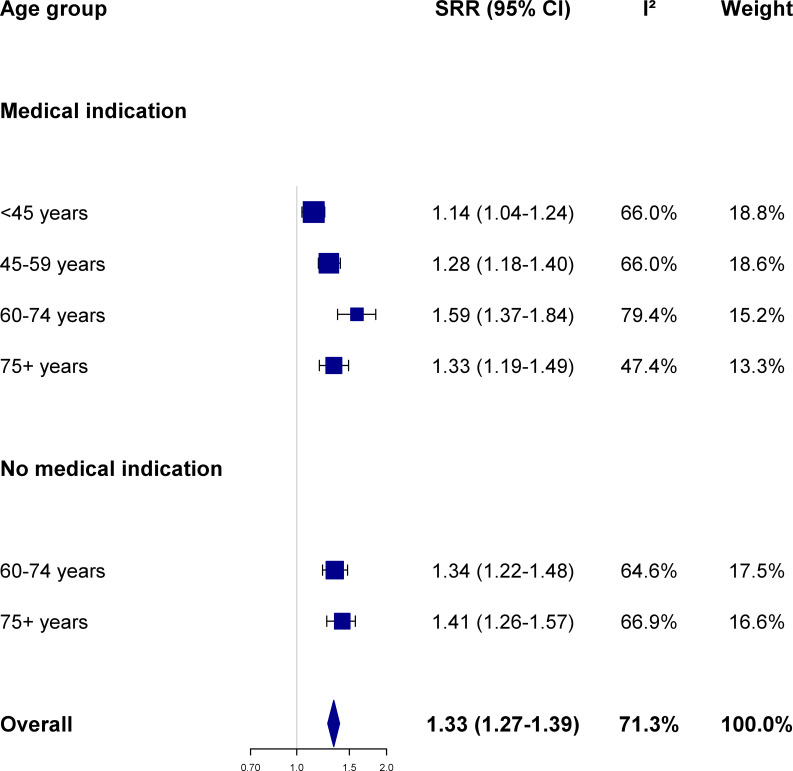
Association between influenza vaccination and the risk of acute respiratory infection according to age group and presence of medical indication. Summary risk ratio (SRR) and 95% confidence intervals (CI) were calculated by pooling season-specific risk ratio using random effects meta-analysis models; between-seasons heterogeneity was quantified using the I^2^ statistics (see text for details; season-specific risk ratio were provided in S2 Table). The Netherlands, seasons 2006/07 to 2015/16.

**Fig 3:**
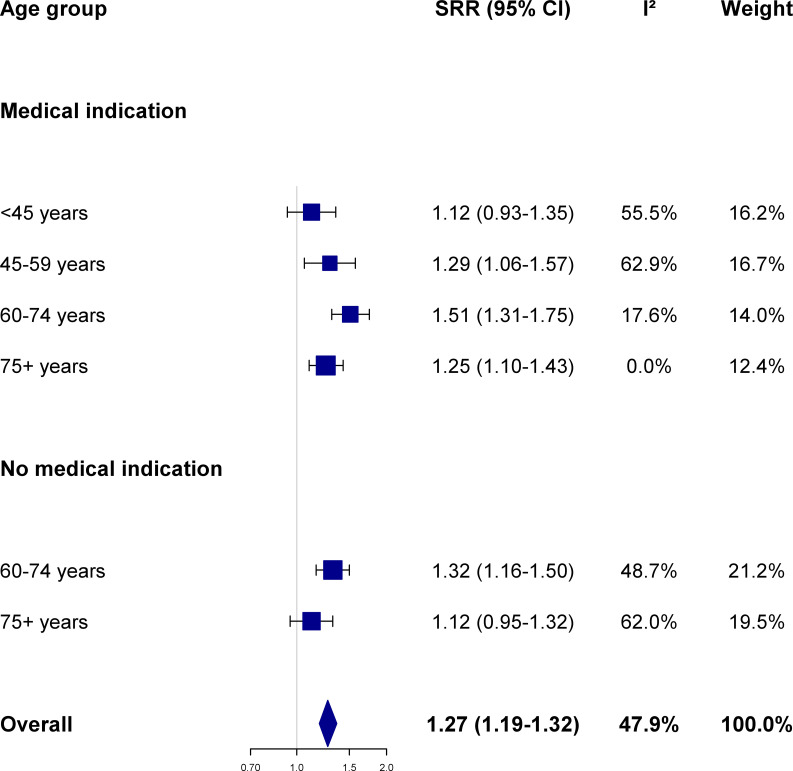
Association between influenza vaccination and the risk of pneumonia according to age group and presence of medical indication. Summary risk ratio (SRR) and 95% confidence intervals (CI) were calculated by pooling season-specific risk ratio using random effects meta-analysis models; between-seasons heterogeneity was quantified using the I^2^ statistics (see text for details; season-specific risk ratio were provided in S3 Table). The Netherlands, seasons 2006/07 to 2015/16.

The influenza vaccine was significantly (p-value <0.001) associated with an increased risk of being diagnosed by one’s GP with each of the four study outcomes. In detail, the risk to be clinically diagnosed with influenza, ARI, pneumonia, and lower back pain among influenza vaccinees was higher by 24%, 33%, 27% and 21%, respectively, overall during the study period compared to subjects who were not vaccinated (Figs 1–3 and S1 Fig). For each of those four conditions, the SRR differed somewhat across age groups and between older participants with or without a medical indication to vaccination.

Overall, vaccinated individuals had a significantly increased risk of receiving a clinical (not laboratory-confirmed) diagnosis of influenza compared to non-vaccinated (SRR 1.24, 95%CI 1.12–1.38, p-value <0.001). In stratified analyses, the increase in risk among vaccinees was significantly higher by around 40% compared to non-vaccinees among subjects who were recommended to receive the vaccine solely on the basis of their age, and by ≈40% among subjects aged 75 years or older who also had a medical indication for the vaccine (Fig 1). In contrast, the increase in risk was modest (≤20%) and did not achieve statistical significance among participants aged 74 years or younger who had a medical indication to be vaccinated. Variability of RRs across seasons was considerable (I^2^>50%) among younger (≤59 years) subjects with a medical indication to vaccinate, and among healthy people aged 60–74 years) (Fig 1 and S1 Table), which was not accounted for, however, by any of the variables that were tested in meta-regression and subgroup analyses.

Influenza vaccine status was associated with a significantly increased risk of being diagnosed with ARI by a GP, both in the whole study population (SRR 1.33, 95%CI 1.27–1.39, p-value <0.001) and in each age group- and medical indication-specific subgroup (SRR ranging between 1.14 and 1.59, always achieving statistical significance) (Fig 2). Also for clinically diagnosed ARI, risk estimates varied largely across seasons, as denoted by the I^2^ value generally exceeding (or close to) the 50% threshold (Fig 2 and S2 Table). In meta-regression models, the relative risk of an ARI diagnosis among vaccinees showed a tendency to increase across seasons among individuals aged <45 years (meta-regression p-value: 0.028) and 45–59 years (p-value 0.038), while it tended to decrease over time (p-value 0.051) among those aged ≥75 years who had no medical indication for vaccination. Instead, the risk of an ARI diagnose among influenza vaccinees did not depend on what was the dominant virus (sub)type in a given season, and whether this was mismatched with the viral strain contained in the vaccine.

The risk of having a clinical diagnosis of pneumonia was significantly greater among vaccinated vs. non-vaccinated individuals (SRR 1.27, 95%CI 1.19–1.36, p-value <0.001) (Fig 3). This held true when study participants were stratified according to age and underlying medical conditions, with statistical significance being achieved in four of the six population subgroups (Fig 3). Unlike influenza and ARI, the heterogeneity of risk estimates across seasons was slightly within the limit of acceptable values (I^2^ = 47.9%) in the overall analysis, although the 50% threshold was exceeded in some study strata (Fig 3 and S3 Table). The gap in risk among vaccinated vs. non-vaccinated individuals tended to increase over time among study subjects with no medical indication for the vaccine (p-value 0.064 and 0.027 for those aged 60–74 and ≥75 years, respectively). In addition, the higher the proportion of influenza cases caused by the A(H3N2) virus subtype, the higher the RR of being diagnosed with pneumonia by a GP among vaccinated vs. non-vaccinated study subjects aged 45–74 individuals with a medical indication to the vaccine, while an inverse correlation emerged in the same study strata with the proportion of cases in a season accounted for by the influenza B virus type.

**Fig 4:**
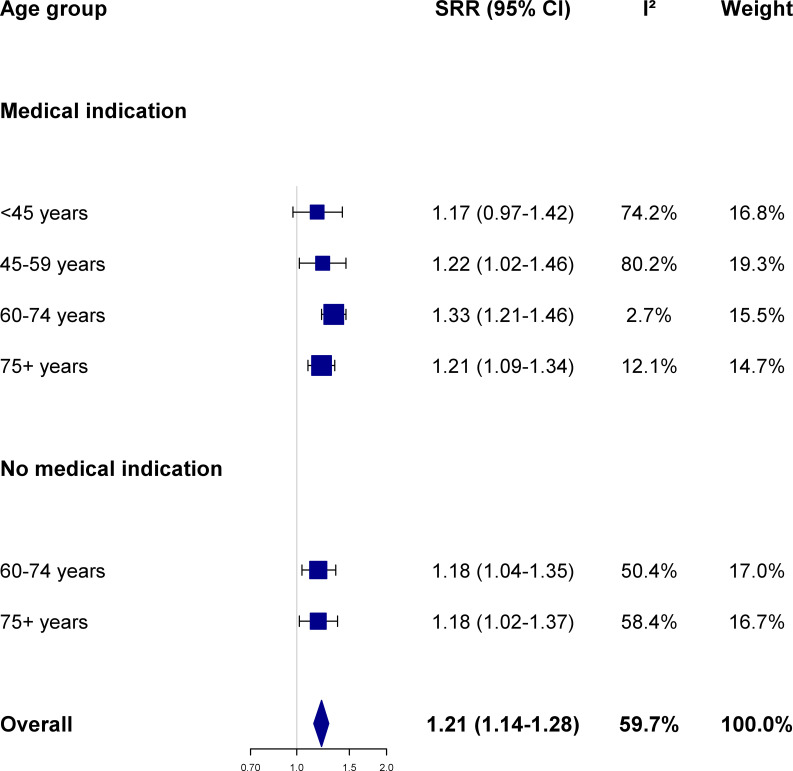
Association between influenza vaccination and the risk of lower back pain according to age group and presence of medical indication. Summary risk ratio (SRR) and 95% confidence intervals (CI) were calculated by pooling season-specific risk ratio using random effects meta-analysis models; between-seasons heterogeneity was quantified using the I^2^ statistics (see text for details; season-specific risk ratio were provided in S4 Table). The Netherlands, seasons 2006/07 to 2015/16.

In addition to influenza, ARI and pneumonia, influenza vaccinees were also significantly more likely to visit their GP because of lower back pain (SRR 1.21, 95%CI 1.14–1.28, p-value <0.001) compared to non-vaccinated study subjects (Fig 4, S4 Table and S1 Text). The SRR comparing the risk of a lower back pain episode among vaccinated vs. unvaccinated individuals did not significantly differ from that for influenza, ARI, or pneumonia in any of the six population strata defined by the subject’ age and health status.

## Discussion

We compared the risk of influenza, ARI, and pneumonia (all of which clinically diagnosed, i.e. not confirmed by laboratory investigations–for influenza and ARI–or X-ray or other tests–for pneumonia), and lower back pain as an independent control, among individuals who were vs. were not vaccinated against influenza in the Netherlands during ten consecutive seasons (from 2006/07 to 2015/16). We found that vaccinated individuals show consistently higher GP consultation rates for all those four health outcomes (an increase of approximately 21–33%). For clinically diagnosed influenza and ARI (but not pneumonia), this was somewhat more evident in the older age groups (60–74 and ≥75 years), while the difference was milder (yet still significant for ARI) and marred by substantial between-seasons heterogeneity among younger individuals with underlying medical conditions. The increase in consultation rates for clinically diagnosed pneumonia tended to be stronger in the seasons dominated by the A(H3N2) influenza virus subtype. Interestingly, GP consultation rates of lower back pain (a health outcome whose incidence was not expected *a priori* to be affected by the vaccine) were also increased among vaccinated individuals (an increase of approximately 21%), and in a comparable fashion to what was observed for influenza, ARI and pneumonia.

As stated in the Introduction, our expectations were that influenza vaccinees would be less likely to receive a clinical diagnosis of influenza, ARI, and pneumonia (although to a lesser extent for the latter two illnesses, which can be caused by several other agents in addition to influenza viruses). Since the findings ran counter to our expectations, we believe it is important to critically examine whether any systematic bias may have been at play. First, a “health seeking behaviour” bias may have been in action, whereby vaccinated individuals are more likely (signs and symptoms of illness being equal) to perceive themselves to have a health problem and, consequently, to seek care by their GP. Another potential bias affecting our study could be labelled as “healthy person” (or “unwell”) bias, whereby those who are more vulnerable to develop a serious illness after a respiratory infection are also more likely to be vaccinated, by their own choice and/or because they are more strongly recommended by their GPs. A further source of bias may originate from GPs themselves, who might be more likely to diagnose any illness, whether preventable or not by the vaccine, while visiting vaccinated individuals. As a partial attempt to discount these sources of bias, we stratified all analyses by age groups and underlying comorbidities (which are plausibly among the strongest determinants of vaccine uptake, health-seeking behaviours, and GPs’ attitude towards their patients), and compared the consultation rates for lower back pain (i.e. a health outcome not expected to be affected by influenza vaccination) with those of clinically diagnosed influenza, ARI, and pneumonia in each of the six study strata. If the vaccine was effective, even in the presence of the above biases, one would expect a milder increase in consultation rates for these clinical diagnoses compared to lower back pain. Instead, we did not observe this in our data, as the increase in GP consultation rates was comparable for all of the outcomes, regardless of whether or not they should be prevented (at least partially) by a truly effective influenza vaccine. Thus, even if residual confounding cannot be ruled out, our data suggest that the influenza vaccine is in reality not associated with a decrease in GP consultation rates for clinically diagnosed influenza, ARI, and pneumonia.

As surprising as they are, our results are consistent with other studies reported in recent years. For instance, the incidence rates of all-cause respiratory illness in children (6 months to 18 years of age) with pre-existing medical conditions [31], and of influenza-like illness among older adults (≥60 years) [32], were shown not to be reduced by influenza vaccination in two recent investigations conducted in the Netherlands. Cowling et al. found an increased risk of non-influenza viral infections of the upper respiratory tract following influenza vaccination in a randomized controlled trial [33], and the prescriptions of respiratory antibiotics was directly associated with receipt of both the inactivated and live-attenuated influenza vaccine in England during 2015–17 [34]. Also, Rifkin et al. reported a post-influenza vaccination increase in ARI among children (but not adults) caused by non-influenza respiratory pathogens [35]. Overall, there seems to be some evidence, although sparse so far and requiring confirmation, that the influenza vaccine may indeed be associated with an increased incidence of respiratory illnesses, possibly caused by other respiratory pathogens (i.e. viruses and bacteria), that are filling the gap left by reduced influenza infection.

The strengths of our study derive from it being population-based and relying on data relating to multiple health outcomes collected over ten consecutive influenza seasons. As already stated in the Introduction, the method we propose and exemplified here aims to complement the results originating from TND studies (whose objective is mainly to estimate the influenza VE), having some important advantages over the latter, e.g. population-based, timelier, less expensive, and easily reproducible in other countries provided EMRs are available (although some differences could arise from the diverse general organization of the health system across countries). Our study has also several limitations that are important to acknowledge. As already mentioned, influenza, ARI and pneumonia were diagnosed clinically, with no subsequent laboratory confirmation, thus some degree of outcome misclassification (possibly differential according to the exposure) is likely to have occurred. A mild misclassification of the exposure (i.e. vaccine status) may also have taken place in the younger age groups, who do not receive the vaccine from their GP but via their employer (2%) [36]. As discussed above, multiple possible source of bias and confounding might have been at play and although we took countermeasures to mitigate their impact on the results, the degree to which these were effective is difficult to assess, and residual confounding cannot be ruled out. Since the biases described above can originate from both patients and GPs, expanding the investigation to include hospitalization and mortality caused by the different health outcomes may provide a further sensitivity analysis to test the robustness of our findings. A given episode of any of the health outcomes in our study was considered as new (and entered in the analyses) if the patient had not visited the GP for the same diagnosis during the last 4 weeks (influenza), 8 weeks (ARI), 16 weeks (pneumonia), or 52 weeks (LBP) [21]. While this time-window seems appropriate for influenza, ARI, and pneumonia (given the natural course of these acute infections), it may be too short for lower back pain, which may instead consist of recurrent symptomatic episodes occurring against the background of a chronic illness. Thus, the number of new lower back pain episodes (and, therefore, of the corresponding GP consultation rates) might have been somewhat exaggerated, in turn causing a relative underestimation of the vaccine’s effect on the other health outcomes. A further limitation is that the information on the proportion of cases caused by each virus sub(type) used in meta-regression models originated from cumulated sentinel and non-sentinel surveillance data, instead of from sentinel surveillance only. Subgroup analyses and meta-regression yielded only modestly significant p-values, and considering the lack of a priori hypothesis and the large number of comparisons that were made, they should be considered as exploratory analyses. Although not a limitation, it is finally worth highlighting that unlike other European countries, conditions representing an indication to vaccinate in the Netherlands do not include severe obesity, pregnancy, and living in a long-term care facility [37]. Therefore, replicating our analyses elsewhere would be helpful to assess the impact on the results of this and other aspects that are specific of each country. These may include, for instance, the general structure of the healthcare system (in particular, of the primary care sector), which may influence people’s care-seeking behaviors and ultimately the results of the method we propose here.

In conclusion, we found that, contrary to expectations, influenza vaccinees in all ten winters received a clinical diagnosis of influenza, ARI and pneumonia from their GP more often than unvaccinated individuals, and that this increase in risk was similar in magnitude to that observed for other health conditions not affected by the vaccine, e.g. lower back pain. Importantly, since the data are not laboratory confirmed, our results should not be linked directly to influenza, but rather to respiratory illnesses in general. The finding on lower back pain suggests that our findings on clinically diagnosed respiratory illnesses may be at least partially caused by confounding. In addition, our findings suggest that randomized controlled trials implemented to assess new influenza vaccines (e.g. high-dose), which currently have laboratory confirmed influenza as their primary outcome, should also look at other clinical outcomes, including clinically diagnosed and laboratory confirmed cases of ARI and pneumonia (both primary influenza viral and secondary bacterial or fungal), in order to have a more comprehensive picture of the overall health impact of the influenza vaccine.

## Supporting information

S1 Fig (TIFF)

S1 File (DOC)

S1 Text Association between influenza vaccination and lower back pain GP consultations. (DOCX)

S1 Table Association between influenza vaccination and the risk of influenza according to age group and presence of medical indication. Season-specific risk ratio (RR) and 95% confidence intervals (CI); summary risk ratio (SRR) and 95% CI calculated using random effects meta-analysis models; and between-seasons heterogeneity quantified using the I^2^ statistics. The Netherlands, seasons 2006/07 to 2015/16.
(DOCX)

S2 Table Association between influenza vaccination and the risk of acute respiratory infection according to age group and presence of medical indication. Season-specific risk ratio (RR) and 95% confidence intervals (CI); summary risk ratio (SRR) and 95% CI calculated using random effects meta-analysis models; and between-seasons heterogeneity quantified using the I^2^ statistics. The Netherlands, seasons 2006/07 to 2015/16.
(DOCX)

S3 Table Association between influenza vaccination and the risk of pneumonia according to age group and presence of medical indication. Season-specific risk ratio (RR) and 95% confidence intervals (CI); summary risk ratio (SRR) and 95% CI calculated using random effects meta-analysis models; and between-seasons heterogeneity quantified using the I^2^ statistics. The Netherlands, seasons 2006/07 to 2015/16.
(DOCX)

S4 Table Association between influenza vaccination and the risk of lower back pain according to age group and presence of medical indication. Season-specific risk ratio (RR) and 95% confidence intervals (CI); summary risk ratio (SRR) and 95% CI calculated using random effects meta-analysis models; and between-seasons heterogeneity quantified using the I^2^ statistics. The Netherlands, seasons 2006/07 to 2015/16.
(DOCX)
