# Supplementary material for: Impact of influenza vaccination in the Netherlands, 2007–2016: Vaccinees consult their general practitioner for clinically diagnosed influenza, acute respiratory infections, and pneumonia more often than non-vaccinees
Source: PLoS One. 2021 May 28;16(5):e0249883. doi: 10.1371/journal.pone.0249883 (PMC8162646; doi:10.1371/journal.pone.0249883)
Supplement: S1 File — (DOC) [file pone.0249883.s002.doc]

Link: <https://doi.org/10.17026/dans-x5v-5mvv>
