# Supplementary material for: Impact of influenza vaccination in the Netherlands, 2007–2016: Vaccinees consult their general practitioner for clinically diagnosed influenza, acute respiratory infections, and pneumonia more often than non-vaccinees
Source: PLoS One. 2021 May 28;16(5):e0249883. doi: 10.1371/journal.pone.0249883 (PMC8162646; doi:10.1371/journal.pone.0249883)
Supplement: S1 Text — (DOCX) [file pone.0249883.s003.docx]

Influenza vaccinees were more likely to visit their GP because of lower back pain (SRR 1.21, 95%CI 1.14-1.28) than non-vaccinated study subjects (Figure 4). The increase in risk was fairly comparable across patients’ age and comorbidities-defined strata, with the point estimate ranging between 1.17 and 1.33, and statistical significance being always achieved or closely approached (Figure 4 and Supplementary Table 4). Except among older individuals (aged ≥60 years) with medical indications to receive the vaccine, the heterogeneity of risk estimates was always above the 50% threshold. However, this heterogeneity in RR estimates was not explained by any of the influenza epidemic-related variables that were tested in meta-regression models.
