# Supplementary material for: Impact of influenza vaccination in the Netherlands, 2007–2016: Vaccinees consult their general practitioner for clinically diagnosed influenza, acute respiratory infections, and pneumonia more often than non-vaccinees
Source: PLoS One. 2021 May 28;16(5):e0249883. doi: 10.1371/journal.pone.0249883 (PMC8162646; doi:10.1371/journal.pone.0249883)
Supplement: S1 Table — Season-specific risk ratio (RR) and 95% confidence intervals (CI); summary risk ratio (SRR) and 95% CI calculated using random effects meta-analysis models; and between-seasons heterogeneity quantified using the I2 statistics. The Netherlands, seasons 2006/07 to 2015/16. (DOCX) [file pone.0249883.s004.docx]

| **Age group** | **Season** | | | | | | | | | | **Meta-analysis** | | |
| --- | --- | --- | --- | --- | --- | --- | --- | --- | --- | --- | --- | --- | --- |
|  | **2006/07** | **2007/08** | **2008/09** | **2009/10** | **2010/11** | **2011/12** | **2012/13** | **2013/14** | **2014/15** | **2015/16** | **SRR (95%CI)** | **I^2^** |  |
| **Subjects with medical indications to vaccination** | | | | | | | | | | | | | |
| **<45 years** |  |  |  |  |  |  |  |  |  |  |  |  |  |
| RR | 1.77 | 0.59 | 1.11 | 0.59 | 1.10 | 1.36 | 1.74 | 0.82 | 1.55 | 0.93 | **1.13** | **61.7%** |  |
| 95% CI | 0.52-5.96 | 0.13-2.70 | 0.46-2.68 | 0.34-1.03 | 0.60-2.01 | 0.76-2.45 | 1.31-2.31 | 0.52-1.30 | 1.20-2.02 | 0.69-1.25 | **0.88-1.45** |  |  |
| **45-59 years** |  |  |  |  |  |  |  |  |  |  |  |  |  |
| RR | 0.73 | 1.06 | 0.91 | 1.71 | 0.95 | 1.49 | 1.31 | 0.81 | 1.94 | 0.90 | **1.20** | **69.8%** |  |
| 95% CI | 0.12-4.67 | 0.21-5.48 | 0.44-1.90 | 0.95-3.10 | 0.50-1.80 | 0.84-2.63 | 0.98-1.76 | 0.54-1.23 | 1.58-2.37 | 0.68-1.19 | **0.91-1.57** |  |  |
| **60-74 years** |  |  |  |  |  |  |  |  |  |  |  |  |  |
| RR | 2.08 | 3.33 | 1.44 | 0.72 | 3.96 | 0.78 | 1.39 | 0.76 | 1.67 | 1.23 | **1.03** | **15.3%** |  |
| 95% CI | 0.34-12.55 | 0.71-15.77 | 0.70-2.96 | 0.27-1.96 | 2.66-5.91 | 0.44-1.39 | 1.04-1.85 | 0.51-1.13 | 1.35-2.07 | 0.96-1.58 | **0.87-1.22** |  |  |
| **75+ years** |  |  |  |  |  |  |  |  |  |  |  |  |  |
| RR | 1.02 | 1.76 | 1.64 | 1.00 | 3.76 | 2.47 | 1.13 | 1.33 | 1.35 | 1.44 | **1.29** | **0.0%** |  |
| 95% CI | 0.18-5.79 | 0.14-22.68 | 0.45-5.99 | 0.32-3.14 | 0.94-15.12 | 1.15-5.30 | 0.72-1.77 | 0.75-2.33 | 1.01-1.82 | 0.95-2.19 | **1.04-1.60** |  |  |
| **Subjects without medical indications to vaccination** | | | | | | | | | | | | | |
| **60-74 years** |  |  |  |  |  |  |  |  |  |  |  |  |  |
| RR | 6.97 | 0.26 | 1.30 | 1.35 | 0.82 | 1.41 | 1.03 | 0.81 | 1.11 | 0.88 | **1.40** | **80.2%** |  |
| 95% CI | 1.25-38.81 | 0.03-2.14 | 0.51-3.32 | 0.45-4.00 | 0.36-1.86 | 0.77-2.58 | 0.74-1.44 | 0.51-1.30 | 0.89-1.39 | 0.65-1.20 | **1.01-1.93** |  |  |
| **75+ years** |  |  |  |  |  |  |  |  |  |  |  |  |  |
| RR | 4.74 | 0.40 | 1.86 | 1.92 | 1.39 | 1.66 | 1.31 | 0.94 | 1.47 | 1.04 | **1.39** | **0.0%** |  |
| 95% CI | 0.09-256.62 | 0.00-38.41 | 0.55-6.29 | 0.24-15.48 | 0.39-4.97 | 0.75-3.69 | 0.75-2.31 | 0.54-1.64 | 1.04-2.06 | 0.64-1.69 | **1.15-1.67** |  |  |
