# Supplementary material for: Impact of influenza vaccination in the Netherlands, 2007–2016: Vaccinees consult their general practitioner for clinically diagnosed influenza, acute respiratory infections, and pneumonia more often than non-vaccinees
Source: PLoS One. 2021 May 28;16(5):e0249883. doi: 10.1371/journal.pone.0249883 (PMC8162646; doi:10.1371/journal.pone.0249883)
Supplement: S2 Table — Season-specific risk ratio (RR) and 95% confidence intervals (CI); summary risk ratio (SRR) and 95% CI calculated using random effects meta-analysis models; and between-seasons heterogeneity quantified using the I2 statistics. The Netherlands, seasons 2006/07 to 2015/16. (DOCX) [file pone.0249883.s005.docx]

| **Age group** | **Season** | | | | | | | | | | | **Meta-analysis** | | |
| --- | --- | --- | --- | --- | --- | --- | --- | --- | --- | --- | --- | --- | --- | --- |
|  | **2006/07** | **2007/08** | **2008/09** | **2009/10** | **2010/11** | **2011/12** | **2012/13** | **2013/14** | **2014/15** | **2015/16** | **SRR (95%CI)** | | **I^2^** |  |
| **Subjects with medical indications to vaccination** | | | | | | | | | | | | | | |
| **<45 years** |  |  |  |  |  |  |  |  |  |  |  | |  |  |
| RR | 1.21 | 0.71 | 0.92 | 0.88 | 0.97 | 1.27 | 1.22 | 1.27 | 1.28 | 1.22 | **1.14** | | **66.0%** |  |
| 95% CI | 0.86-1.70 | 0.52-0.98 | 0.67-1.27 | 0.67-1.14 | 0.80-1.19 | 1.10-1.46 | 1.09-1.37 | 1.14-1.42 | 1.16-1.40 | 1.09-1.36 | **1.04-1.24** | |  |  |
| **45-59 years** |  |  |  |  |  |  |  |  |  |  |  | |  |  |
| RR | 1.18 | 0.93 | 1.07 | 1.11 | 1.00 | 1.56 | 1.34 | 1.31 | 1.48 | 1.37 | **1.28** | | **66.0%** |  |
| 95% CI | 0.80-1.75 | 0.67-1.30 | 0.79-1.45 | 0.86-1.45 | 0.81-1.23 | 1.36-1.79 | 1.19-1.50 | 1.18-1.47 | 1.35-1.61 | 1.22-1.53 | **1.18-1.40** | |  |  |
| **60-74 years** |  |  |  |  |  |  |  |  |  |  |  | |  |  |
| RR | 1.27 | 1.03 | 1.44 | 1.11 | 1.78 | 1.41 | 1.21 | 1.16 | 1.50 | 1.46 | **1.59** | | **79.4%** |  |
| 95% CI | 0.83-1.96 | 0.71-1.48 | 1.02-2.01 | 0.81-1.51 | 1.41-2.24 | 1.20-1.65 | 1.06-1.37 | 1.03-1.30 | 1.36-1.64 | 1.30-1.64 | **1.37-1.84** | |  |  |
| **75+ years** |  |  |  |  |  |  |  |  |  |  |  | |  |  |
| RR | 2.84 | 2.01 | 1.26 | 1.68 | 1.09 | 1.58 | 1.25 | 1.33 | 1.28 | 1.27 | **1.33** | | **47.4%** |  |
| 95% CI | 1.90-4.24 | 1.36-2.97 | 0.89-1.79 | 1.19-2.38 | 0.83-1.42 | 1.32-1.88 | 1.09-1.44 | 1.16-1.52 | 1.15-1.43 | 1.11-1.45 | **1.19-1.49** | |  |  |
| **Subjects without medical indications to vaccination** | | | | | | | | | | | | | | |
| **60-74 years** |  |  |  |  |  |  |  |  |  |  |  | |  |  |
| RR | 1.78 | 1.29 | 1.39 | 2.61 | 1.40 | 2.44 | 1.31 | 1.51 | 1.36 | 1.45 | **1.34** | | **64.6%** |  |
| 95% CI | 1.05-3.02 | 0.79-2.10 | 0.93-2.08 | 1.79-3.79 | 1.02-1.93 | 2.04-2.92 | 1.13-1.53 | 1.31-1.74 | 1.22-1.53 | 1.26-1.68 | **1.22-1.48** | |  |  |
| **75+ years** |  |  |  |  |  |  |  |  |  |  |  | |  |  |
| RR | 1.96 | 1.28 | 2.14 | 2.52 | 1.05 | 1.06 | 1.33 | 1.30 | 1.37 | 1.25 | **1.41** | | **66.9%** |  |
| 95% CI | 0.96-3.99 | 0.72-2.26 | 1.35-3.41 | 1.49-4.24 | 0.74-1.50 | 0.84-1.33 | 1.11-1.59 | 1.10-1.53 | 1.20-1.56 | 1.06-1.47 | **1.26-1.57** | |  |  |
