# Supplementary material for: Impact of influenza vaccination in the Netherlands, 2007–2016: Vaccinees consult their general practitioner for clinically diagnosed influenza, acute respiratory infections, and pneumonia more often than non-vaccinees
Source: PLoS One. 2021 May 28;16(5):e0249883. doi: 10.1371/journal.pone.0249883 (PMC8162646; doi:10.1371/journal.pone.0249883)
Supplement: S3 Table — Season-specific risk ratio (RR) and 95% confidence intervals (CI); summary risk ratio (SRR) and 95% CI calculated using random effects meta-analysis models; and between-seasons heterogeneity quantified using the I2 statistics. The Netherlands, seasons 2006/07 to 2015/16. (DOCX) [file pone.0249883.s006.docx]

| **Age group** | **Season** | | | | | | | | | | **Meta-analysis** | |
| --- | --- | --- | --- | --- | --- | --- | --- | --- | --- | --- | --- | --- |
|  | **2006/07** | **2007/08** | **2008/09** | **2009/10** | **2010/11** | **2011/12** | **2012/13** | **2013/14** | **2014/15** | **2015/16** | **SRR (95%CI)** | **I^2^** |
| **Subjects with medical indications to vaccination** | | | | | | | | | | | | |
| **<45 years** |  |  |  |  |  |  |  |  |  |  |  |  |
| RR | 0.32 | 0.49 | 0.91 | 0.94 | 0.96 | 0.93 | 1.18 | 1.23 | 1.60 | 1.31 | **1.12** | **55.5%** |
| 95% CI | 0.10-1.06 | 0.19-1.28 | 0.43-1.93 | 0.44-2.03 | 0.57-1.60 | 0.67-1.29 | 0.90-1.56 | 0.93-1.62 | 1.31-1.96 | 1.02-1.68 | **0.93-1.35** |  |
| **45-59 years** |  |  |  |  |  |  |  |  |  |  |  |  |
| RR | 0.59 | 1.12 | 1.64 | 0.45 | 0.97 | 1.77 | 1.24 | 1.83 | 1.50 | 1.13 | **1.29** | **62.9%** |
| 95% CI | 0.23-1.50 | 0.37-3.44 | 0.76-3.56 | 0.20-1.00 | 0.56-1.65 | 1.22-2.57 | 0.96-1.61 | 1.45-2.32 | 1.25-1.80 | 0.90-1.42 | **1.06-1.57** |  |
| **60-74 years** |  |  |  |  |  |  |  |  |  |  |  |  |
| RR | 0.68 | 0.86 | 0.98 | 1.36 | 0.84 | 1.65 | 1.31 | 1.60 | 1.42 | 1.33 | **1.51** | **17.6%** |
| 95% CI | 0.36-1.29 | 0.44-1.69 | 0.52-1.86 | 0.72-2.57 | 0.53-1.31 | 1.27-2.13 | 1.06-1.61 | 1.35-1.91 | 1.22-1.64 | 1.12-1.58 | **1.31-1.75** |  |
| **75+ years** |  |  |  |  |  |  |  |  |  |  |  |  |
| RR | 0.37 | 0.42 | 1.28 | 1.31 | 0.88 | 1.05 | 1.22 | 1.41 | 1.35 | 1.22 | **1.25** | **0.0%** |
| 95% CI | 0.17-0.79 | 0.20-0.89 | 0.68-2.43 | 0.63-2.70 | 0.54-1.43 | 0.78-1.41 | 0.97-1.53 | 1.17-1.71 | 1.15-1.57 | 1.00-1.49 | **1.10-1.43** |  |
| **Subjects without medical indications to vaccination** | | | | | | | | | | | | |
| **60-74 years** |  |  |  |  |  |  |  |  |  |  |  |  |
| RR | 3.42 | 1.88 | 2.50 | 1.49 | 1.60 | 2.01 | 1.25 | 1.68 | 1.58 | 1.15 | **1.32** | **48.7%** |
| 95% CI | 1.21-9.67 | 0.55-6.43 | 0.87-7.20 | 0.63-3.56 | 0.82-3.11 | 1.25-3.23 | 0.91-1.71 | 1.27-2.21 | 1.29-1.95 | 0.88-1.50 | **1.16-1.50** |  |
| **75+ years** |  |  |  |  |  |  |  |  |  |  |  |  |
| RR | 1.91 | 1.07 | 3.18 | 1.30 | 1.03 | 1.76 | 1.26 | 1.40 | 1.11 | 1.20 | **1.12** | **62.0%** |
| 95% CI | 0.29-12.76 | 0.33-3.46 | 0.86-11.82 | 0.46-3.72 | 0.50-2.14 | 1.03-3.01 | 0.89-1.78 | 1.05-1.87 | 0.88-1.39 | 0.88-1.63 | **0.95-1.32** |  |
