# Supplementary material for: Impact of influenza vaccination in the Netherlands, 2007–2016: Vaccinees consult their general practitioner for clinically diagnosed influenza, acute respiratory infections, and pneumonia more often than non-vaccinees
Source: PLoS One. 2021 May 28;16(5):e0249883. doi: 10.1371/journal.pone.0249883 (PMC8162646; doi:10.1371/journal.pone.0249883)
Supplement: S4 Table — Season-specific risk ratio (RR) and 95% confidence intervals (CI); summary risk ratio (SRR) and 95% CI calculated using random effects meta-analysis models; and between-seasons heterogeneity quantified using the I2 statistics. The Netherlands, seasons 2006/07 to 2015/16. (DOCX) [file pone.0249883.s007.docx]

| **Age group** | **Season** | | | | | | | | | | | **Meta-analysis** | | |
| --- | --- | --- | --- | --- | --- | --- | --- | --- | --- | --- | --- | --- | --- | --- |
|  | **2006/07** | **2007/08** | **2008/09** | **2009/10** | **2010/11** | **2011/12** | **2012/13** | **2013/14** | **2014/15** | **2015/16** | **SRR (95%CI)** | | **I^2^** |  |
| **Subjects with medical indications to vaccination** | | | | | | | | | | | | | | |
| **<45 years** |  |  |  |  |  |  |  |  |  |  |  | |  |  |
| RR | 1.41 | 0.79 | 0.83 | 2.42 | 0.72 | 1.09 | 0.95 | 1.09 | 1.35 | 1.59 | **1.17** | | **74.2%** |  |
| 95% CI | 0.68-2.91 | 0.37-1.69 | 0.45-1.55 | 1.58-3.70 | 0.50-1.05 | 0.82-1.45 | 0.74-1.21 | 0.91-1.30 | 1.14-1.61 | 1.31-1.95 | **0.97-1.42** | |  |  |
| **45-59 years** |  |  |  |  |  |  |  |  |  |  |  | |  |  |
| RR | 5.97 | 0.88 | 0.99 | 1.29 | 1.12 | 1.02 | 1.04 | 1.27 | 1.08 | 1.09 | **1.22** | | **80.2%** |  |
| 95% CI | 3.56-9.99 | 0.50-1.53 | 0.59-1.66 | 0.82-2.02 | 0.83-1.53 | 0.81-1.28 | 0.85-1.26 | 1.09-1.48 | 0.93-1.25 | 0.93-1.29 | **1.02-1.46** | |  |  |
| **60-74 years** |  |  |  |  |  |  |  |  |  |  |  | |  |  |
| RR | 0.98 | 0.74 | 1.05 | 1.26 | 1.00 | 1.89 | 1.17 | 1.12 | 1.25 | 1.06 | **1.33** | | **2.7%** |  |
| 95% CI | 0.47-2.01 | 0.39-1.39 | 0.54-2.01 | 0.74-2.14 | 0.70-1.43 | 1.46-2.44 | 0.93-1.47 | 0.94-1.35 | 1.06-1.47 | 0.87-1.28 | **1.21-1.46** | |  |  |
| **75+ years** |  |  |  |  |  |  |  |  |  |  |  | |  |  |
| RR | 0.43 | 1.09 | 1.67 | 0.99 | 1.26 | 1.81 | 1.12 | 1.20 | 1.09 | 1.08 | **1.21** | | **12.1%** |  |
| 95% CI | 0.20-0.94 | 0.52-2.27 | 0.91-3.06 | 0.58-1.69 | 0.83-1.91 | 1.41-2.32 | 0.90-1.39 | 1.00-1.43 | 0.93-1.29 | 0.89-1.31 | **1.09-1.34** | |  |  |
| **Subjects without medical indications to vaccination** | | | | | | | | | | | | | | |
| **60-74 years** |  |  |  |  |  |  |  |  |  |  |  | |  |  |
| RR | 1.25 | 1.46 | 1.03 | 1.31 | 1.52 | 1.05 | 1.63 | 1.31 | 1.46 | 1.16 | **1.18** | | **50.4%** |  |
| 95% CI | 0.56-2.80 | 0.69-3.11 | 0.53-1.97 | 0.70-2.43 | 0.98-2.37 | 0.80-1.37 | 1.28-2.06 | 1.08-1.60 | 1.22-1.75 | 0.93-1.46 | **1.04-1.35** | |  |  |
| **75+ years** |  |  |  |  |  |  |  |  |  |  |  | |  |  |
| RR | 1.05 | 0.71 | 0.92 | 1.83 | 1.81 | 0.94 | 1.27 | 1.19 | 1.32 | 1.16 | **1.18** | | **58.4%** |  |
| 95% CI | 0.41-2.66 | 0.32-1.58 | 0.45-1.88 | 0.93-3.58 | 1.09-3.02 | 0.70-1.26 | 0.98-1.63 | 0.96-1.46 | 1.10-1.58 | 0.94-1.45 | **1.02-1.37** | |  |  |
